# Metabolic Regulation of Hypoxia-Inducible Transcription Factors: The Role of Small Molecule Metabolites and Iron

**DOI:** 10.3390/biomedicines6020060

**Published:** 2018-05-17

**Authors:** Peter S. J. Bailey, James A. Nathan

**Affiliations:** Cambridge Institute for Medical Research, Department of Medicine, University of Cambridge, Cambridge CB2 0XY, UK

**Keywords:** hypoxia inducible factors, HIF, prolyl hydroxylase, PHD, 2-OG dependent dioxygenase, 2-hydroxyglutarate, 2-HG, iron metabolism, TCA cycle

## Abstract

Hypoxia-inducible transcription factors (HIFs) facilitate cellular adaptations to low-oxygen environments. However, it is increasingly recognised that HIFs may be activated in response to metabolic stimuli, even when oxygen is present. Understanding the mechanisms for the crosstalk that exists between HIF signalling and metabolic pathways is therefore important. This review focuses on the metabolic regulation of HIFs by small molecule metabolites and iron, highlighting the latest studies that explore how tricarboxylic acid (TCA) cycle intermediates, 2-hydroxyglutarate (2-HG) and intracellular iron levels influence the HIF response through modulating the activity of prolyl hydroxylases (PHDs). We also discuss the relevance of these metabolic pathways in physiological and disease contexts. Lastly, as PHDs are members of a large family of 2-oxoglutarate (2-OG) dependent dioxygenases that can all respond to metabolic stimuli, we explore the broader role of TCA cycle metabolites and 2-HG in the regulation of 2-OG dependent dioxygenases, focusing on the enzymes involved in chromatin remodelling.

## 1. Introduction

Hypoxia-Inducible transcription Factors (HIFs) underpin metazoan cellular responses to oxygen availability [1,2,3,4,5], coordinating transcriptional changes that allow cells to adapt to hypoxic environments. However, HIFs may also be activated independently of oxygen through a multitude of mechanisms, including mitochondrial function, changes in intracellular metabolites or iron availability, reactive oxygen species (ROS) generation, and modulation of the mammalian target of rapamycin (mTOR) pathway. This review focuses on the role of small molecule metabolites and iron in altering HIF activity.

HIFs are a set of basic helix-loop-helix transcription factors, comprising an α regulatory subunit (HIF-1α, HIF-2α or HIF-3α) and a constitutively expressed β counterpart (HIF-β, also known as aryl hydrocarbon nuclear translocation factor, ARNT). In aerobic conditions, the HIF-1α and HIF-2α subunits are rapidly degraded by proteasomes [6,7], preventing the formation of the dimeric HIF complex (the role of HIF3α in the HIF response remains unclear). Key to this rapid degradation are the Prolyl Hydroxylase Domain containing enzymes (PHDs, also known as EGLNs), which act as oxygen sensors.

When oxygen is present, PHDs hydroxylate two conserved proline residues within the oxygen-dependent degradation domain (ODD) of HIF-α (used to refer to both HIF-1 and HIF-2 α-isoforms) [8,9]. Prolyl hydroxylated HIF-α is then recognised for degradation by the Von Hippel Lindau (VHL) ubiquitin E3 ligase complex, which targets the transcription factor for rapid proteasome-mediated degradation [6,7]. When oxygen tension is low, HIF-α is no longer prolyl-hydroxylated, and is stabilised, whereupon it can heterodimerise with HIF-β, translocate to the nucleus, and activate HIF responsive genes through binding to HIF response elements (HRE). Genetic studies suggest that hundreds of genes are associated with the HRE consensus sequence [10,11,12], covering a multitude of pathways including glucose metabolism, cell signalling and adhesion, and angiogenesis. The clinical importance of this hypoxia response pathway is highlighted by the role of HIFs in diverse cellular processes, such as the modulation of inflammation and immune responses [13,14,15], stem cell differentiation and tumour formation [16,17]. Indeed, VHL loss-of-function mutations are found in up to 90% of all renal cell carcinomas [18,19].

PHDs are members of a large family of over 60 2-oxoglutarate (2-OG, also known as α-ketoglutarate) dependent dioxygenases [20], and thus their activity is affected by the abundance of 2-OG (a key tricarboxylic acid (TCA) cycle metabolite) and ferrous iron. When oxygen, 2-OG, and ferrous iron are present, PHDs oxidatively decarboxylate 2-OG to produce succinate and carbon dioxide [21]. Therefore, as well as being highly sensitive to oxygen concentrations, PHDs can be considered as both oxygen and metabolic sensors.

In this review, we describe recent developments in the understanding of the metabolic control of the HIF response by small molecule metabolites and iron. We first explore how PHDs can be regulated by TCA cycle intermediates and 2-OG derivatives, and then examine the relevance of this metabolic HIF response in physiological and disease settings. Lastly, we discuss the interplay between this metabolic regulation of HIFs and other 2-OG-dependent dioxygenases in cell fate decisions, and how exploring the pathways involved may present new opportunities to intervene therapeutically in HIF-dependent disease processes.

## 2. Regulation of HIFs by TCA Cycle Metabolites

**Figure 1:**
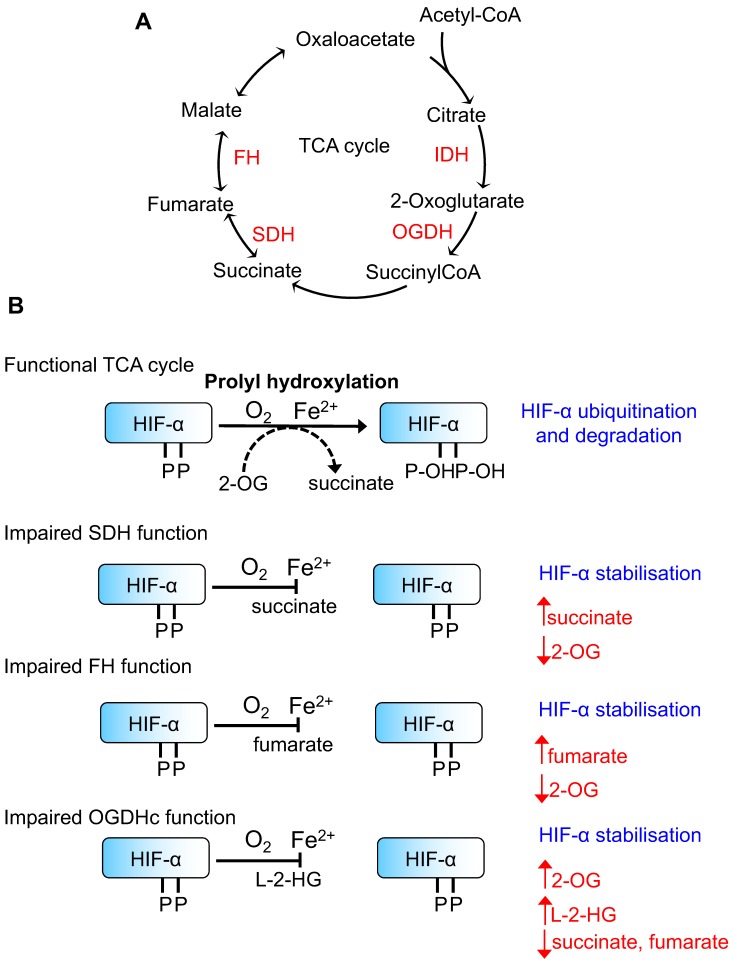
Tricarboxylic acid (TCA) cycle metabolites and Hypoxia-Inducible transcription Factor (HIF)-α prolyl hydroxylation. (**A**) Schematic of the TCA cycle illustrating key enzymes involved in altering the HIF response. (**B**) Schematic of HIF-α prolyl hydroxylation by Prolyl Hydroxylase Domain containing enzymes (PHDs). When the TCA cycle is functional, HIF-α is prolyl hydroxyated which acts as the signal for Von Hippel Lindau (VHL)-mediated ubiquitination and subsequent proteasome-mediated degradation. The effects of different TCA cycle enzyme mutations/loss of function on key metabolites and prolyl hydroxylation are shown. IDH = isocitrate dehydrogenase, OGDHc = 2-oxoglutarate dehydrogenase complex, SDH = succinate dehydrogenase, FH = fumarate hydratase.

The first observations that metabolite regulation of HIFs may be of relevance to disease stemmed from studies examining hereditary cancer syndromes arising from mutations in genes involved in TCA cycle function and mitochondrial respiration (Figure 1). Mutations in the succinate dehydrogenase (SDH) subunits (mainly SDHB and SDHD) lead to familial paraganglioma syndromes [22,23], and it has been noted that some of these tumours resemble the hypoxia-induced carotid body enlargement observed in individuals living at high altitudes [23]. Moreover, paragangliomas in patients with SDHD mutations have increased levels of HIF-2α and angiogenic factors [24], and the transcriptional profile of phaeochromocytomas with mutations in SDH subunits is similar to those seen in VHL-mutant tumours [25]. Interestingly, hereditary heterozygous mutations in another TCA cycle enzyme, fumarate hydratase (FH), also lead to the development of renal cell carcinomas following the loss of heterozygosity, similarly to VHL or SDH mutations [26,27]. Moreover, HIF-1α and HIF-2α levels have been observed to accumulate in tumours with FH mutations [28,29], implying that the accumulation of fumarate is linked to HIF activation.

The most plausible explanation for the activation of the HIF response in these familial cancer syndromes relates to altered PHD activity due to the accumulation of TCA cycle small molecule metabolites. Consistent with this hypothesis, Selak et al. [30] and Isaacs et al. [28] demonstrated that the HIF-α stabilisation, observed in SDH or FH loss, can be reversed in cells following the addition of 2-OG. However, it should be noted that SDH mutations also affect the function of the electron transport chain, which could also alter mitochondrial ROS formation and oxygen availability for PHD activity.

Based on these hereditary tumour syndrome observations, several groups have determined the levels of TCA cycle intermediates that would be required to directly inhibit PHD activity. Hewitson et al. [31] assayed the effect and structural basis for the inhibition of PHD activity by TCA cycle intermediates using a truncated form of human PHD2, and a synthetic peptide derived from the HIF-1α oxygen-dependent degradation domain. PHD2 contains a long, disordered region at its N-terminus (residues 1–187), and thus, most enzymatic assays and structures published involve constructs where this has been removed. Using this truncated PHD2, Hewitson et al. found that succinate and fumarate compete with 2-OG at the active site iron atom, forming a complex which is then unable to bind with the HIF peptide. Through this and other studies, estimates for the affinities of succinate and fumarate, as well as other TCA cycle intermediates, to the PHD isoforms have been determined [31,32,33,34] (Table 1). Fumarate has the highest affinity for PHDs, with PHD2 being most susceptible to competitive inhibition by this metabolite. However, it is important to consider that the relative abundance of 2-OG will also influence PHD activity and the binding of these enzymes to HIFs, as recently demonstrated by Abboud et al. [35].

## 3. Regulation of HIFs by 2-Hydroxyglutarate

Aside from the regulation of prolyl hydroxylation by TCA cycle intermediates, it is becoming clear that other 2-OG derivatives can also modulate HIF function through competitive inhibition of PHD activity (Figure 1). 

The reduced form of 2-OG, 2-hydroxyglutarate (2-HG), is a chiral compound that can be detected in both enantiomeric forms, d(r)-2-HG and l(s)-2-HG, in cells. While this is unusual for metabolites, as stereoisomerism is key to physiological enzymatic processes, the identification of d-2-HG and l-2-HG specific dehydrogenases (D2HGDH [37] and L2HGDH [38]) in mammals indicates that both enantiomers have biological roles. Furthermore, human germline homozygous mutations in D2HGDH or L2HGDH lead to d- or l-2-hydroxyglutaric aciduria, (rare metabolic syndromes involving delayed development, seizures, progressive brain damage, and death usually in early childhood), consistent with both 2-HG enantiomers having physiological roles [39]. One explanation for these findings is that 2-HG enantiomers are unwanted by-products of oxidative metabolism that must be oxidised by their respective dehydrogenases to prevent mitochondrial dysfunction. Alternatively, 2-HG enantiomers may act as signalling molecules and have distinct metabolic functions. This latter hypothesis is supported by the identification that patients with homozygous germline mutations in both L2HGDH and D2HGDH are predisposed to brain tumours [40,41], and recent studies have implicated 2-HG enantiomers in tumorigenesis and cell fate decisions through their actions on 2-OG dependent dioxygenases (also discussed in Section 6).

### 3.1. d-2-HG and the HIF Response

Mutations of both isocitrate dehydrogenase isoforms (IDH1 and IDH2) are linked to tumorigenesis and are detected in acute myeloid leukaemia, colorectal cancers, and in a large proportion of high grade gliomas and glioblastomas [42,43]. Most cancer-associated IDH mutations occur in the catalytic active site, leading to neomorphic activity, whereby the enzyme reduces 2-oxoglutarate to d-2-HG, coupled by NADPH [44]. In IDH1 (the cytosolic NADP-dependent isoform), almost all gliomas are associated with an active site R132H mutation [45], whereas in IDH2 (the mitochondrial NAD-dependent homolog), the main active site mutation is R172K [44].

Tumour cells expressing mutant IDH1/2 enzymes have very high levels of d-2-HG (100-fold greater than non-IDH mutant tumours) [46], and some display constitutive activation of HIF target genes [47]. The ability of d-2-HG to compete with 2-OG binding to several 2-OG dependent dioxygenases involved in chromatin remodelling is thought to promote tumorigenesis (discussed in Section 6). However, whether HIFs are stabilised or degraded in the context of IDH mutations and d-2-HG remains an area of debate.

In astrocytes, where HIF-1α appears to have tumour-suppressor effects, d-2-HG production has clearly been shown to stimulate PHD2-mediated degradation of HIF, enhancing cell proliferation [48]. However, HIFs have also been reported to be constitutively active in mutant IDH glioblastomas. Zhao et al. [49] observed that overexpression of IDH1 R132H mutants in cultured cells had an inhibitory effect on wildtype IDH, leading to a decrease in available 2-OG, and that the HIF stabilisation could be reversed by the addition of exogenous 2-OG. They hypothesised that decreased PHD2 activity results from reduced availability of 2-OG. However, more recent metabolomic studies of mutant IDH1 gliomas have suggested that 2-OG is rapidly replaced by glutaminolysis [50], and it is therefore unlikely that depletion of 2-OG could account for the loss of PHD activity seen.

It is possible that the PHD inhibition seen in some d-2-HG producing mutant IDH-cell types may be an indirect effect. Sasaki et al. [51] reported that the brains of transgenic mice expressing mutant IDH1 had markedly elevated NADP/NADPH ratios (consistent with mutant IDH catalysing reduction of 2-OG to d-2-HG and increasing the cellular NADP pool), with an associated decrease in reduced glutathione (GSH) and ascorbate. They hypothesised that the depletion of ascorbate may lead to inhibition of 2-OG dependent dioxygenases, and therefore, HIF activation.

In vitro assays support the observations in cells that d-2-HG may increase PHD activity [36], consistent with the findings that d-2-HG can promote HIF degradation [48]. Tarhonskaya et al. [52] showed that in vitro activity of PHD2 can be activated by d-2-HG, in an iron and ascorbate dependent mechanism, where endogenous 2-OG is reoxidised from d-2-HG via a non-enzymatic Fenton reaction with the active site iron. Thus, in contrast to findings involving the TCA cycle intermediates [31], most studies do not support a major role for d-2-HG in inhibiting PHDs and instead demonstrate that PHD2 activity can be increased.

### 3.2. l-2-HG Activates a HIF Response in Hypoxia or Following the Accumulation of 2-OG

The biological functions of l-2-HG had remained obscure until recently, when it was noted that l-2-HG accumulates in hypoxic conditions. Two groups independently reported that this accumulation of l-2-HG occurred when 2-OG increased in cells [53,54]. This l-2-HG formation was not mediated by IDH, but by the non-canonical activity of lactate dehydrogenase (LDHA) and the malate dehydrogenases (MDH1/2), coupled to the oxidation of NADH [20]. The accumulation of l-2-HG altered the activity of 2-OG dependent dioxygenases involved in chromatin remodelling, but did not alter the HIF response [54]. Prior in vitro studies have suggested that relatively high levels of l-2-HG would be required to inhibit PHD2 activity (IC_50_ of ≈ 400 µM) [36]; thus, the concentrations seen in these hypoxia studies may not have additive effects on the already suppressed PHD activity.

However, using a unbiased gene-trap mutagenesis screen in human cells, we identified that l-2-HG accumulation is associated with HIF activation, independently of oxygen availability [55]. Disruption of the 2-oxoglutarate dehydrogenase complex (OGDHc) stabilised HIF-1α levels in aerobic conditions due to decreased PHD activity [55]. We found that impaired OGDHc function increased 2-OG levels, leading to the formation of l-2-HG by LDHA/MDH, which inhibited PHD2 [55]. Moreover, increasing the levels of 2-OG in cells (i.e., with exogenous cell permeable 2-OG, dimethyl 2-OG) was sufficient to promote l-2-HG formation, PHD inhibition and HIF activation [55,56]. Nadtochiy et al. [57] proposed that an acidic pH is important for altering the catalytic activity of LDHA and MDH to promote l-2-HG formation; their findings were subsequently confirmed by Intlekofer et al. [56]. 

Tyrakis et al. [58] provided further evidence for a physiological role of l-2-HG in hypoxia, demonstrating that l-2-HG can modulate both PHD activity and 2-OG dependent dioxygenases involved in chromatin remodelling. Hypoxic accumulation of l-2-HG in CD8+ T lymphocytes drove T cell differentiation, dependent on whether the activity of both HIF and histone demethylases was altered [58]. Interestingly, MDH depletion had no effect on l-2-HG formation in the lymphocytes. Instead, l-2-HG production was dependent on HIF-1α-induced upregulation of pyruvate dehydrogenase kinase, leading to the inhibition of pyruvate dehydrogenase, thereby promoting glutaminolysis as the carbon source for the TCA cycle. This metabolic reprogramming increased levels of 2-OG which could then act as a substrate for NADH-coupled reduction to l-2-HG by LDHA.

Together these studies highlight an emerging role for l-2-HG in altering the activity of PHDs in aerobic and hypoxic conditions. It will be of interest in future studies to explore whether l-2-HG alters the activity of other PHD enzymes similarly to PHD2 and to understand if acidic cellular environments, such as those that occur in solid tumours, promote l-2-HG formation. It is also noteworthy that other small molecule metabolites may be directly or indirectly implicated in the activation of the HIF response. For example, in triple negative breast cancer cells, glutamate secretion results in inhibition of the xCT glutamine–cystine transporter, reduction of intracellular cysteine, and oxidative self-inactivation of PHDs [59]. The potential effect of metabolite levels and amino acid abundance on mTOR activity, which will also alter HIF-α abundance [60], must also be taken in account, adding further complexity to the metabolic regulation of HIFs.

## 4. Mitochondrial Lipoylation and the HIF Response

The identification that disrupting the function of the 2-oxoglutarate dehydrogenase complex (OGDHc) inhibits PHD activity also led to the discovery that mitochondrial lipoylation can influence the HIF response [55].

**Figure 2:**
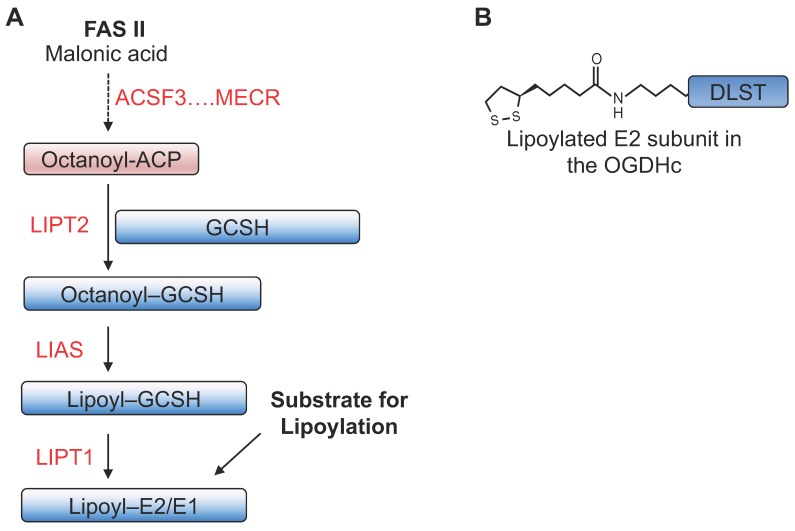
Mitochondrial protein lipoylation. (**A**) Schematic of the pathway involved in the formation of lipoylated proteins, highlighting some of the enzymes involved. Octanoyl-acyl carrier protein (ACP) is formed from FAS II. Acyl-CoA Synthetase Family Member 3 (ACSF3) is thought to be the first enzyme involved in this pathway. Mitochondrial enoyl-CoA reductase (MECR) is involved in the formation of acyl-ACP. Lipoylated substrates consist of the E1 and E2 subunits of the pyruvate dehydrogenase complex, the E2 subunit of the branched chain ketoacid dehydrogenase complex, and the H subunit of the glycine cleavage system (GCSH). GCSH is thought to be first octanoylated by Lipoyl(Octanoyl) Transferase 2 (LIPT2), and lipoic acid synthase (LIAS) then catalyses the formation of lipoate from the octanoylated protein. LIPT1 is thought to be involved in the transfer of the lipoate moieties to the other E1/E2 subunits. (**B**), The lipoyl moiety of dihydrolipoamide succinyltransferase (DLST) within the 2-oxoglutarate dehydrogenase complex (OGDHc) is shown.

The OGDHc forms a rate-limiting step in the TCA cycle and is important in controlling the flux of amino acids into the cycle [61]. Its E2 subunit is one of five mitochondrial enzymes that require lipoylation for catalytic activity (along with the E1 and E2 subunits of the pyruvate dehydrogenase complex, the E2 subunit of the branched chain ketoacid dehydrogenase complex, and the H subunit of the glycine cleavage system) [62] (Figure 2). The OGDHc enzymatic reaction is as follows: (1) the triamine pyrophosphate group in the E1 subunit, OGDH, attacks 2-oxoglutarate, removing carbon dioxide to form a carbanionic intermediate; (2) this reacts with the lipoate group on the E2 subunit, DLST (dihydrolipoamide succinyltransferase) (Figure 2), reducing the lipoate to dihydrolipoate and forming a succinyl intermediate which is transferred to coenzyme A; and finally (3) the E3 subunit, DLD (dihydrolipoamide dehydrogenase), oxidises the dihydrolipoate of the E2 subunit back to lipoate, passing its electrons to NADH [63]. Most eukaryotes require lipoate to be synthesised de novo, in a process dependent on the mitochondrial fatty acid synthesis pathway and lipoic acid synthase (LIAS) [62].

Our gene-trap mutagenesis approach identified that mutations in LIAS leads to decreased OGDHc, l-2-HG formation, PHD inhibition and HIF-1α stabilisation [55]. Moreover, we observed similar findings in fibroblasts derived from patients with germline mutations in LIAS [55], which clinically manifests as a variant form of Leigh syndrome [62,64]. The full biological implications for the regulation of PHD activity by lipoylation remain to be determined, but it is possible that HIF activation in the variant-form of Leigh syndrome may function as a survival mechanism to counteract the impaired mitochondrial function due to LIAS loss. For instance, in a CRISPR/Cas9 knockout genome-wide screen to look for genes that promote survival when the mitochondrial respiratory chain is disrupted, Jain et al. identified that VHL loss promotes cell survival and went on to show that PHD inhibition or hypoxia improves survival in mouse models of Leigh syndrome [65].

Subsequently, Paredes et al. also observed that disruption of OGDHc lipoylation can lead to HIF-1α stabilisation [66]. Knockdown of polymerase δ interacting protein 2 (POLDIP2), a nuclear-encoded protein of uncertain function [67], leads to stabilisation of HIF and loss of lipoylation of the OGDHc E2 subunit. Surprisingly, Paredes et al. found reduced levels of 2-OG in POLDIP2-depleted cells, which is in contrast to most studies on inhibition of OGDHc function [53,54,55,56]. However, their assay relied on colorimetric measurements of 2-OG using an alanine transferase-dependent assay, where it is possible that the depletion of 2-OG levels reported simply represent inhibition of the enzyme due to the presence of l-2-HG. 

While the loss of lipoate results in HIF-1α stabilization, disrupting mitochondrial fatty acid synthesis (FAS II) upstream of lipoylation appears to have the opposite effect on HIF-1α. Depletion of mitochondrial enoyl-CoA reductase (MECR) resulted in decreased HIF-1α levels in 3T3 cells following incubation in 1% oxygen [68], but mitochondrial lipoylation was not measured. The authors suggest that both ROS and 2-OG levels may be implicated in this increased turnover of HIF-1α (for detailed reviews on ROS and HIFs please see [69,70,71]).

Lastly, the recent identification of Sirtuin 4 (SIRT4) as a mitochondrial lipoamidase raises the possibility that mitochondrial lipoylated enzymes may be dynamically regulated [72,73], and that this may influence the activity of PHDs or other 2-OG dependent dioxygenases. SIRT4 removes lipoate from the pyruvate dehydrogenase complex in vitro and when overexpressed [72,73]. The role of SIRT4 in OGDHc regulation and the HIF axis has not yet been examined.

## 5. HIFs and Iron Metabolism

The links between oxygen and iron metabolism have been apparent since initial observations that hypoxia was associated with iron absorption in rodent models [74,75] and that iron-dependent PHDs regulate HIFα stability. There is now considerable evidence that both HIF-1α and HIF-2α can activate genes involved in iron regulation, including transferrin [76], the transferrin receptor [77], heme-oxgenase [78], ferroportin [79], erythropoietin (EPO) and hepcidin [80] (for further details on the relative role of HIF-1α versus HIF-2α in iron metabolism please see detailed reviews such as Simpson et al. [81] and Peyssonnaux et al. [82]). Moreover, the importance of iron levels in directly altering PHD activity is demonstrated by the ability of membrane-impermeable ferric iron (Fe^3+^) chelators (e.g., desferrioxamine (DFO)) to promote HIFα stabilisation [83,84], and by observations that iron deficiency is associated with exaggerated acute hypoxic pulmonary hypertension [85]. Given this close association of iron with HIF activity, it is of interest to explore whether changes in the iron labile pool are implicated in the regulation of HIFα stability.

Most iron is transported into cells in the ferric form bound to transferrin. Antibody-mediated transferrin receptor inhibition or depletion of the receptor itself activates an HIF response [86,87], highlighting the importance of transferrin-mediated iron uptake for PHD activity. Intracellular iron binding proteins may also be important for delivering iron to newly synthesised PHD molecules. Nandal et al. [88] showed that the cytosolic iron chaperone, PCBP1, is required for PHD2 activity, and that its depletion leads to stabilisation of HIFα. The role of intracellular iron stores (e.g., ferritin) in modulating HIF activity is less clear. In dendritic cells, HIF-1α promotes inflammatory signalling in response to bacterial lipopolysaccharides (LPS) [89], and it has been proposed that this is, in part, driven by NF-κB-related induction of ferritin formation, leading to depletion of intracellular iron [90]. However, in iron replete conditions, we have not observed that the prevention of ferritin breakdown (an autophagic process termed ferritinophagy) alters PHD activity [87]. It is possible that when iron availability is reduced, ferritin may be an important source of iron for PHD function.

In addition to transferrin uptake and ferritin breakdown, proteins involved in reducing ferric iron to the ferrous form and trafficking intracellular iron may also alter PHD activity and HIF responses. Inhibition or depletion of the Vacuolar ATPase (V-ATPase), the main proton pump for acidifying endolysosomal compartments, stabilises HIFα by depleting the labile iron pool [87]. HIFs are activated within 2 h of V-ATPase inhibition [87], suggesting that ferroreductase activity is important for maintaining the ferrous iron required for PHD activity. Prolonged V-ATPase inhibition will also lead to impaired transferrin uptake (by impaired clathrin-mediated endocytosis) [91] and ferritinophagy [92], which may result in further decreases in the cellular iron content. However, which ferroreductases are important for PHD activity remains to be determined.

The ability of changes in intracellular iron content to alter PHD activity is quite remarkable when one considers that in most iron-dependent enzymes, iron is integral to the structure of the protein. In vitro assays of PHD2 activity have revealed that purified PHD2 active site domains have a strong affinity for ferrous iron, (Kd < 1 µM) [93], yet cellular and animal studies of PHD2 function have revealed an apparently reversible phenotype whereby PHD2 activity can be restored in iron deplete conditions by supplementation with ferrous iron. Whether this reversible phenotype is due to newly synthesised PHD molecules or is dependent on the delivery of ferrous iron to the PHD active site is not known.

## 6. Metabolic Control of PHDs and Other 2-OG Dependent Dioxygenases in Disease

While PHDs are central to controlling HIFα stability and the hypoxia response, it is important to consider that other 2-OG dependent dioxygenases, which have diverse cellular functions, may also respond to changes in oxygen or metabolite availability. Here, we highlight the role of three families of 2-OG dependent dioxygenases which are of particular relevance to the HIF response: Factor Inhibiting HIF (FIH), Ten-Eleven Translocation (TET) methylcytosine dioxygenases, and Jumonji Histone DeMethylases (JHDMs).

### 6.1. Regulation of Factor Inhibiting HIF by Small Molecule Metabolites

FIH is a 2-OG dependent dioxygenase which hydroxylates an asparagine residue in the C-terminal transactivation domains of HIF-1α and HIF-2α [94], blocking the recruitment of p300 and thus, suppressing the transcription of oxygen-inducible genes. This is oxygen sensitive, but with a lower K_m_, such that it is active at lower oxygen concentrations than PHD2 [95]. As well as its role in regulating HIF, FIH also has broader asparagine hydroxylase activity [96], targeting proteins involved in Notch signalling [97,98], NF-κB signalling [99], and ubiquitination [100,101].

Similarly to PHDs, FIH activity can be altered by TCA cycle intermediates, but in vitro studies show a more specific pattern of inhibition. Citrate and oxaloacetate have an IC_50_ for FIH hydroxylation of an HIF peptide within physiological concentrations (0.6 and 1.2 mM, respectively [34]), whereas fumarate and succinate do not appear to inhibit this asparagine hydroxylase (IC_50_ > 10 mM) [34]. Interestingly, l-2-HG has been shown to inhibit FIH at similar concentrations to PHD2 (IC_50_ <1 mM) [36]. Thus, it is possible that FIH inhibition may contribute to L-2-HG mediated upregulation of HIF target genes in hypoxia.

### 6.2. Metabolic Control of TETs and JHDMs

Metabolism can alter chromatin in diverse ways, but particularly relevant to this discussion are the roles of small molecule metabolites on DNA and histone methylation by the TETs and JHDMs.

TET methylcytosine dioxygenases catalyse the hydroxylation of 5-methylcytosine. 5-hydroxymethylcytosine is then further oxidised by the same enzymes to 5-formylcytosine and 5-carboxycytosine, which are rapidly excised by thymidine DNA glycosylase and replaced by unmodified cytosines [102], leading to demethylation of chromosomal DNA. This DNA demethylation has important functional consequences for genomic imprinting, the repression of transposable elements, and the regulation of transcription [103].

JHDMs are a structurally diverse family of more than 30 2-OG dependent dioxygenases [104], which catalyse the removal of methyl moieties from the Nε side chain of lysine residues in histones This likely occurs via an initial Nε-methyl group hydroxylation. The resulting hemiaminal intermediate is unstable, fragmenting into demethylated lysine and formaldehyde [104]. Demethylation alters the structure of chromatin, with important consequences for transcriptional regulation and genome stability. For example, trimethylated lysine 4 of histone 3 (H3K4me3) is a marker of transcriptional start sites and regulatory elements, whereas H3K9me3 and H3K27me3 are associated with transcriptional repression [105]. 

An example of the interplay between HIF activity, DNA demethylation and histone demethylation in driving disease processes is apparent in neuroendocrine tumours with SDH mutations. As previously discussed, somatic and germline mutations of SDH subunits are commonly identified in patients with paraganglioma [22,106,107], where the accumulation of succinate inhibits HIF prolyl hydroxylation, driving the expression of HIF target genes [30]. However, paragangliomas with SDH mutations also display markedly abnormal patterns of DNA hypermethylation. Letouze et al. [108] found distinct clusters of DNA methylation in paragangliomas with mutations in different SDH subunits. Similar findings were observed in patients with FH germline mutations [108]. Interestingly, these tumours showed particular susceptibility to treatment with inhibitors of DNA methlyation [108].

Similar to the PHD enzymes, succinate and fumarate inhibit TET demethylases at concentrations that are observed in hereditary cancer syndromes [33], and this is likely to be implicated in the development of these tumours. Recent work by Sciacovelli et al. demonstrated that FH mutations and fumarate accumulation inhibit TET activity by promoting epithelial-to-mesenchymal-transition (EMT), which is associated with cancer initiation, invasion, and metastasis [109].

It is important to note that, as well as affecting the function of 2-OG dioxygenases, the elevations in succinate or fumarate observed in SDH or FH hereditary cancer syndromes may have additional consequences for cellular function. For example, FH mutations alter aconitase activity through succination [110] and disruption of SDH activity leads to extensive changes in histone succinylation patterns, which correlate with changes in gene expression [111].

### 6.3. 2-Hydroxyglutarate and Cancers

Although d-2-hydroxyglutarate is a poor inhibitor of PHDs, accumulation of this metabolite in IDH-mutant tumours has marked effects on chromatin remodelling. Firstly, global changes in DNA methylation have been observed in acute myeloid leukaemia (AML), with a specific subset of methylation patterns corresponding to tumours expressing mutant IDH [112]. Identical aberrances in DNA methylation are seen in AML cells with mutations in the TET2 DNA demethylase. Secondly, Lu et al. [113] observed that the expression of mutant IDH1 or IDH2 in cell lines or patient-derived cells was associated with marked increases in histone methylation, independent of changes in DNA methylation. This was replicated by exogenous addition of d-2-HG.

There remains, however, a paradox between the effects of the two 2-HG enantiomers in the development of cancers, much like that of their differential effect on HIF stabilisation. Exogenous delivery of the D enantiomer is in itself sufficient to drive proliferation of leukaemic cell lines, yet delivery of l-2-HG has no effect on cell growth [114]. In contrast, in vitro studies have suggested that the L enantiomer is a far more potent inhibitor of all 2-OG dependent dioxygenases [115]. The explanation for these findings remains uncertain. It is possible that concomitant stabilisation of HIF in conditions of l-2-HG accumulation is protective, as Losman et al. [114] showed that knockdown of PHD2 in mutant IDH1-expressing cell lines restored their differentiation.

### 6.4. l-2-Hydroxyglutarate in Cell Fate Decisions

The formation of l-2-HG in hypoxia has global effects on DNA and histone demethylation. Intlekofer et al. [54] observed that increases in total H3K9 trimethylation in glioblastoma cell lines induced by hypoxia could be attenuated by overexpression of L2HGDH [38], raising the possibility that the accumulation of l-2-HG in hypoxia might have direct and synergistic effects on JHDMs in epigenetic regulation. Tyrakis et al. [58] provided further evidence for a potential physiological role of l-2-HG. Treating cultured CD8+ lymphocytes with l-2-HG in normoxia led to increased expression of CD62L and other T cell differentiation markers, changes which persisted in lymphocytes from HIF-1α or HIF-2α knockout mice. Genetic manipulation of endogenous l-2-HG by either knockout or overexpression of L2HGDH led to significant changes in CD8+ T lymphocyte differentiation markers. This was associated with changes in histone H3K27 trimethylation and in DNA methylation, consistent with modulation of JHDM and TET activity.

Anso et al. [116] demonstrated the potential signalling implications of l-2-HG accumulation in haematopoietic cell fate decisions. Haematopoietic stem cells, despite being highly glycolytic cells with low mitochondrial mass, were highly susceptible to the knockout of mitochondrial complex III, which led to loss of their ability to differentiate, and a global increase in DNA and histone methylation. Unbiased metabolite analysis revealed elevated l-2-HG levels, as well as a markedly decreased NAD/NADH ratio. Anso et al. concluded that the disruption of mitochondrial complex III causes a reduced NAD/NADH ratio, thereby favouring reduction of 2-OG into l-2-HG [116]. Li and colleagues also observed that l-2-HG may alter cell fate determination in *Drosophila* larvae [117]. In this case l-2-HG is synthesized in aerobic glycolysis through the upregulation of *Drosophila* LDH (dLDH) and inhibition of dL2HGDH in a lactate-dependent manner [117].

Together, these studies indicate that l-2-HG levels influence cell phenotypes, which may be of importance in both physiological and disease settings. However, the relative importance of l-2-HG to the oxygen-mediated regulation of 2-OG-dependent dioxygenases remains unclear. In particular, whether l-2-HG accumulates in distinct subcellular compartments will be important to determine.

## 7. Conclusions and Future Directions

The advent of unbiased metabolite profiling technologies and advances in the interpretation of broad-spectrum metabolomics has offered important opportunities for the study of metabolic regulators of HIFs. However, little is understood about the relevance of metabolite transport between cell compartments and how this subcellular localisation impacts signalling via 2-OG dependent dioxygenases. In addition, the affinities of different 2-OG dependent dioxygenases for oxygen, iron and 2-OG are likely to have marked implications for modulating their activity in disease and physiological settings. Advances in localised metabolomics, such as the ability to isolate mitochondria for metabolite profiling [118], allow more detailed analysis of metabolite fluxes. However, the rapid movement of metabolites between compartments still makes these types of approaches challenging. Novel imaging modalities, such as hyperpolarised Magnetic Resonance Imaging (MRI) [119], now offer the ability to assess metabolite fluxes on a whole organism level, and these techniques will provide important new insight into the role of small molecule metabolites in altering cell fates. Alongside these technical developments, a deeper understanding of the relative importance of metabolic stimuli compared to oxygen sensitivity for the activity of individual 2-OG dependent dioxygenases may delineate cellular pathways where inhibition of the enzymes may be of therapeutic benefit.

Lastly, given the recent application of PHD inhibitors and HIF antagonists in the clinical setting, it will be important to explore the effect of small molecule metabolites on these drugs. Several PHD inhibitors are in phase III clinical trials for anaemia related to chronic kidney disease [120], and crystallographic studies predict that they bind to the active site metal and 2-OG to inhibit catalytic activity [121]. Therefore, 2-OG and the levels of other small molecule metabolites may influence the activity of these inhibitors, and such interactions may have important consequences for diseases where 2-OG metabolism is impaired. It is possible the association of TCA cycle intermediates or 2-HG with 2-OG dependent dioxygenases may offer insight into new approaches for developing drugs to target these enzymes.

## Figures and Tables

**Table 1:** Published IC_50_ values of TCA cycle metabolites and 2-hydroxyglutarate (2-HG) enantiomers as inhibitors of HIF prolyl hydroxylases PHD1, PHD2 and PHD3.

Metabolite	PHD1 IC_50_	PHD2 IC_50_	PHD3 IC_50_
Oxaloacetate [34]	1 mM	3.8 mM	1.2 mM
Citrate [34]	6.3 mM	4.8 mM	550 µM
Succinate [34]	830 µM	510 µM	570 µM
Fumarate [34]	120 µM	80 µM	60 µM
l-2-hydroxyglutarate [36]		420 µM	
d-2-hydroxyglutarate [36]		7.3 mM	
